# Supplementary material for: Microstructural organization of human insula is linked to its macrofunctional circuitry and predicts cognitive control
Source: eLife. 2020 Jun 4;9:e53470. doi: 10.7554/eLife.53470 (PMC7308087; doi:10.7554/eLife.53470)
Supplement: Supplementary file 1. — It also contains the Microstructural properties of insular subdivisions in the macaque brain in Supplementary Table 3. Finally, the sorted weights of the first component in brain-behavior canonical correlation analysis for the analysis using functional parcellations of the insula (Deen et al., 2011) is included in Supplementary Table 4; and the result of the same analysis using multimodal parcellations of the insula (Glasser et al., 2016) is included in Supplementary Table 5. [file elife-53470-supp1.docx]

Supplementary Tables

**Supplementary Table 1. von Mises-Fisher statistics for the three insula subdivisions dAI, vAI, and PI.** For each functional parcellation-derived subdivision^29^, we computed the gradient’s main direction on each subject. The single subject 95% confidence interval (c.i.) summarizes the confidence intervals obtained across-subjects when computing their gradient’s mean direction. The population’s confidence interval shows the confidence interval for the mean direction computed from the subjects’ main directions. The Rayleigh statistic allows to reject the hypothesis of no preferential direction at the group level. The parcels with a p < 1e-10 where marked with an asterisk.

| **Insula Subdivision** | **Single subject 95% c.i. (angle)** | **Group Level 95% c.i. (angle)** | **Rayleigh statistic** |
| --- | --- | --- | --- |
| Left PI* | 0.22π +/- 0.09π | 0.03 π | 634 |
| Left dAI* | 0.21π +/- 0.09π | 0.03 π | 596 |
| Left vAI* | 0.25π +/- 0.10π | 0.05 π | 252 |
| Right PI* | 0.26π +/- 0.11π | 0.05 π | 170 |
| Right dAI* | 0.24π +/- 0.10π | 0.03 π | 569 |
| Right vAI | 0.27π +/- 0.10π | 0.09 π | 72 |

**Supplementary Table 2. von Mises-Fisher statistics for the three insula subdivisions dAI, vAI, and PI.** For each multimodal parcellation-derived subdivision^35^, we computed the gradient’s main direction on each subject. The single subject 95% confidence interval (c.i.) summarizes the confidence intervals obtained across-subjects when computing their gradient’s mean direction. The population’s confidence interval shows the confidence interval for the mean direction computed from the subjects’ main directions. The Rayleigh statistic allows to reject the hypothesis of no preferential direction at the group level. The parcels with a p < 1e-10 where marked with an asterisk.

| **Insula Subdivision** | **Single subject 95% c.i. (angle)** | **Group Level 95% c.i. (angle)** | **Rayleigh statistic** |
| --- | --- | --- | --- |
| Left PI* | 0.15π +/- 0.06π | 0.02 π | 976 |
| Left dAI* | 0.20π +/- 0.09π | 0.02 π | 838 |
| Left vAI* | 0.24π +/- 0.10π | 0.03 π | 747 |
| Right PI* | 0.22π +/- 0.08π | 0.03 π | 496 |
| Right dAI | 0.25π +/- 0.10π | 0.04 π | 370 |
| Right vAI * | 0.23π +/- 0.10π | 0.03 π | 553 |

**Supplementary Table 3.** **Microstructural properties of insular subdivisions in the macaque brain.**

|  | **Ial versus the rest of the Insular Cortex Regions** | | | | | | | | | | | | | |
| --- | --- | --- | --- | --- | --- | --- | --- | --- | --- | --- | --- | --- | --- | --- |
|  | **X77** | | | | | | | | **X181** | | | | | |
|  | **Left Hemisphere** | | | **Right Hemisphere** | | | | | **Left Hemisphere** | | | **Right Hemisphere** | | |
|  | **t** | **p-value** | **df** | **t** | | **p-value** | **df** | **t** | | **p-value** | **df** | **t** | **p-value** | **df** |
| Ig | -60.91 | < 0.001 | 4570 | -86.54 | | < 0.001 | 4872 | -76.73 | | < 0.001 | 5040 | -76.19 | < 0.001 | 5650 |
| Id | -35.49 | < 0.001 | 4618 | -47.16 | | < 0.001 | 4948 | -52.25 | | < 0.001 | 4584 | -51.91 | < 0.001 | 4729 |
| Iai | -55.04 | < 0.001 | 6573 | -51.60 | | < 0.001 | 8277 | -40.42 | | < 0.001 | 6455 | -36.76 | < 0.001 | 6642 |
| Iapl | -25.47 | < 0.001 | 2402 | -31.76 | | < 0.001 | 2207 | -47.50 | | < 0.001 | 3918 | -47.26 | < 0.001 | 4678 |
| Ia | 2.77 | 0.006 | 616 | -13.41 | | < 0.001 | 409 | 6.11 | | < 0.001 | 733 | -2.89 | 0.004 | 825 |
| Iapm | 0.72 | 0.473 | 1121 | -17.35 | | < 0.001 | 1256 | 10.87 | | < 0.001 | 1644 | -7.79 | < 0.001 | 1354 |
| Iam | -44.28 | < 0.001 | 3933 | -40.28 | | < 0.001 | 2446 | -49.24 | | < 0.001 | 3770 | -59.67 | < 0.001 | 4266 |
|  |  |  |  |  |  | |  |  | |  |  |  |  |  |
|  | **Ig versus the rest of the Insular Cortex Regions** | | | | | | | | | | | | | |
|  | **X77** | | | | | | | | **X181** | | | | | |
|  | **Left Hemisphere** | | | **Right Hemisphere** | | | | | **Left Hemisphere** | | | **Right Hemisphere** | | |
|  | **t** | **p-value** | **df** | **t** | **p-value** | | **df** | **t** | | **p-value** | **df** | **t** | **p-value** | **df** |
| Ial | 60.91 | < 0.001 | 4570 | 86.53 | < 0.001 | | 4872 | 76.73 | | < 0.001 | 5040 | 76.19 | < 0.001 | 5650 |
| Id | 49.61 | < 0.001 | 20062 | 75.61 | < 0.001 | | 20842 | 47.55 | | < 0.001 | 18223 | 48.52 | < 0.001 | 17327 |
| Iai | -1.95 | 0.05 | 8209 | 18.68 | < 0.001 | | 6136 | 44.22 | | < 0.001 | 9880 | 50.63 | < 0.001 | 10979 |
| Iapl | 12.02 | < 0.001 | 1413 | 16.88 | < 0.001 | | 1413 | 18.65 | | < 0.001 | 1909 | 28.04 | < 0.001 | 2533 |
| Ia | 41.03 | < 0.001 | 376 | 20.31 | < 0.001 | | 328 | 63.15 | | < 0.001 | 412 | 60.14 | < 0.001 | 424 |
| Iapm | 33.32 | < 0.001 | 773 | 26.28 | < 0.001 | | 881 | 72.68 | | < 0.001 | 896 | 35.31 | < 0.001 | 959 |
| Iam | 4.75 | < 0.001 | 1703 | 14.20 | < 0.001 | | 1393 | 14.39 | | < 0.001 | 1853 | 6.91 | < 0.001 | 2180 |

**Supplementary Table 4.** **Sorted weights of the first component in brain-behavior canonical correlation analysis. Analysis using functional parcellations of the insula^29^.**

| **RTOP measures** | **Weights** |
| --- | --- |
| rvAI | 0.04 |
| rPI | 0.03 |
| lPI | 0.01 |
| ldAI | -0.003 |
| lvAI | -0.05 |
| rdAI | -0.07 |
|  |  |
| **Behavior measures** | **Weights** |
| Flanker | 0.02 |
| ListSort | 0.01 |
| Gamberling_Perc_Larger | 0.01 |
| WM_ACC | 0.01 |
| WM_RT | 0.01 |
| Relation_RT | 0.006 |
| CardSort | 0.001 |
| ProcSpeed | -0.001 |
| Gambling_RT_Larger | -0.02 |
| Picseq | -0.02 |
| Relation_ACC | -0.04 |

Out-scanner behavioral measures:

“ListSort”, “Flanker”, “CardSort”, “ProcSpeed” and “Picseq” are standardized scores to evaluate individuals’ performance in the corresponding listing sorting, flanker, card sorting, processing speed and picture sequence tasks from the NIH Toolbox (<http://www.healthmeasures.net/explore-measurement-systems/nih-toolbox>).

In-scanner behavioral measures:

“WM_Accuracy” stands for the accuracy in the n-back working memory task.

“WM_RT” stands for the reaction time in the n-back working memory task.

“Gambling_Perc_Larger” stands for the probability to select larger items in the gambling task.

“Gambling_RT_Larger” stands for the reaction time to select larger items in the gambling task.

“Relational_Accuracy” stands for the accuracy in the relational processing task.

“Relational_RT” stands for the reaction time in the relational processing task.

**Supplementary Table 5.** **Sorted weights of the first component in brain-behavior canonical correlation analysis. Analysis using multimodal parcellations of the insula^35^**

| **RTOP measures** | **Weights** |
| --- | --- |
| rvAI | 0.02 |
| rPI | 0.02 |
| lPI | 0.01 |
| lvAI | -0.02 |
| ldAI | -0.02 |
| rdAI | -005 |
|  |  |
| **Behavior measures** | **Weights** |
| WM_RT | 0.02 |
| Flanker | 0.02 |
| WM_ACC | 0.02 |
| ListSort | 0.007 |
| Gambling_Perc_Large | 0.002 |
| ProcSpeed | -0.003 |
| CardSort | -0.005 |
| Relation_RT | -0.01 |
| PicSeq | -0.02 |
| Gambling_RT_Larger | -0.02 |
| Relation_ACC | -0.04 |

Out-scanner behavioral measures:

“ListSort”, “Flanker”, “CardSort”, “ProcSpeed” and “Picseq” are standardized scores to evaluate individuals’ performance in the corresponding listing sorting, flanker, card sorting, processing speed and picture sequence tasks from the NIH Toolbox (<http://www.healthmeasures.net/explore-measurement-systems/nih-toolbox>).

In-scanner behavioral measures:

“WM_Accuracy” stands for the accuracy in the n-back working memory task.

“WM_RT” stands for the reaction time in the n-back working memory task.

“Gambling_Perc_Larger” stands for the probability to select larger items in the gambling task.

“Gambling_RT_Larger” stands for the reaction time to select larger items in the gambling task.

“Relational_Accuracy” stands for the accuracy in the relational processing task.

“Relational_RT” stands for the reaction time in the relational processing task.
